# EMMPRIN regulates tumor growth and metastasis by recruiting bone marrow-derived cells through paracrine signaling of SDF-1 and VEGF

**DOI:** 10.18632/oncotarget.5331

**Published:** 2015-09-22

**Authors:** Yanke Chen, Xingchun Gou, Derek Kai Kong, Xiaofei Wang, Jianhui Wang, Zeming Chen, Chen Huang, Jiangbing Zhou

**Affiliations:** ^1^ Experiment Center of Biomedical Research School of Medicine, Xi'an Jiaotong University, Xi'an 710061, P. R. China; ^2^ Department of Neurosurgery, Yale University, New Haven, CT 06511, USA; ^3^ Laboratory of Cell Biology and Translational Medicine, Xi'an Medical University, Xi'an 710021, P. R. China; ^4^ Department of Pathology, Yale University, New Haven, CT 06511, USA; ^5^ Department of Biomedical Engineering, Yale University, New Haven, CT 06510, USA

**Keywords:** EMMPRIN, bone marrow-derived cells, SDF-1, VEGF, tumor growth and metastasis

## Abstract

EMMPRIN, a cell adhesion molecule highly expressed in a variety of tumors, is associated with poor prognosis in cancer patients. Mechanistically, EMMPRIN has been characterized to contribute to tumor development and progression by controlling the expression of MMPs and VEGF. In the present study, by using fluorescently labeled bone marrow-derived cells (BMDCs), we found that the down-regulation of EMMPRIN expression in cancer cells reduces tumor growth and metastasis, and is associated with the reduced recruitment of BMDCs. Further protein profiling studies suggest that EMMPRIN controls BMDC recruitment through regulating the secretion of soluble factors, notably, VEGF and SDF-1. We demonstrate that the expression and secretion of SDF-1 in tumor cells are regulated by EMMPRIN. This study reveals a novel mechanism by which EMMPRIN promotes tumor growth and metastasis by recruitment of BMDCs through controlling secretion and paracrine signaling of SDF-1 and VEGF.

## INTRODUCTION

Extracellular matrix metalloproteinase inducer (EMMPRIN), also known as CD147, is an evolutionarily conserved member of the adhesion molecule family. EMMPRIN is widely expressed in human cells and plays an important role in many normal physiological functions [1, 2]. EMMPRIN is also highly expressed in a variety of tumors and the over-expression of EMMPRIN is associated with poor prognosis in cancer patients [2–7], suggesting that EMMPRIN may be an important factor that contributes to malignant tumor progression. Mechanistically, it is known that EMMPRIN functions to promote secretion of matrix metalloproteinases (MMPs) [8, 9] and vascular endothelial growth factor (VEGF) [10, 11]. Nonetheless, accumulating evidence suggests that EMMPRIN has multiple roles in tumor development and progression beyond what has been reported. We recently reported that EMMPRIN promotes tumor glycolysis through monocarboxylate transporters (MCTs) [12], and regulates tumor angiogenesis through controlling the expression of insulin-like growth factor-I (IGF-I), in addition to VEGF [13]. Others have reported that EMMPRIN promotes tumor invasion and chemoresistance through stimulation of the urokinase-type plasminogen activator (uPA) system [14] and hyaluronan signaling [15].

Bone marrow-derived cells (BMDCs) play an important role in tumor progression and metastasis. As a component of the tumor stroma, BMDCs promote tumor growth through secretion and paracrine signaling of a variety of growth, survival, and proangiogenic factors, as well as extracellular matrix–modifying enzymes [16]. BMDCs also contribute to tumor progression by incorporating into the blood vessel endothelium as endothelial progenitor cells [17, 18]. During tumor development and progression, BMDCs actively interact with tumor cells. In primary tumors, BMDCs are recruited to the tumor microenvironment, where they promote tumor growth, vascularization, and metastasis [19–21]. In tumor metastases, BMDCs migrate to pre-metastatic sites, where they form cellular clusters prior to the arrival of tumor cells [21, 22].

Although both EMMPRIN and BMDC recruitment are involved in tumor progression and metastasis, their relationship in tumor development has not been explored. In the present study, we investigated the impact of EMMPRIN on BMDCs by studying GFP labeled BMDC donor cells in EMMPRIN down-regulated Lewis lung carcinoma (LLC) tumor xenografts. We found that down-regulation of EMMPRIN expression slowed tumor growth and metastasis, and correlated with a reduced number of BMDCs in the tumor microenvironment. We demonstrated that the recruitment of BMDCs is mediated through soluble factors, mainly stromal cell-derived factor 1 (SDF-1) and VEGF, and the expression and secretion of SDF-1 are directly controlled by EMMPRIN. Our results suggest a novel mechanism by which EMMPRIN promotes tumor progression.

## RESULTS

### Down-regulation of EMMPRIN expression slows tumor progression, and is correlated with decreased BMDC clusters

**Figure 1:**
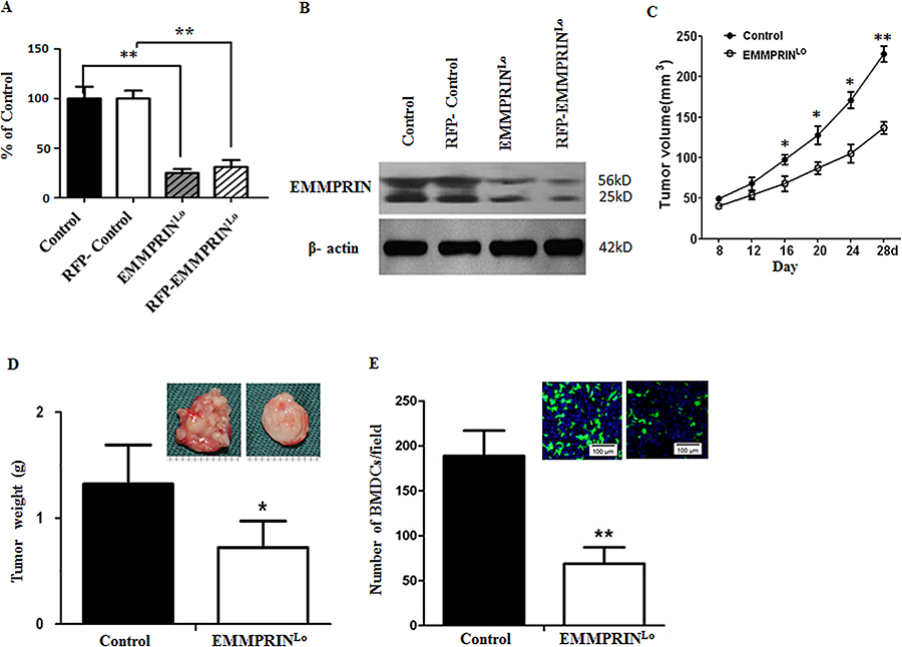
Down-regulation of EMMPRIN reduces tumor growth and BMDC recruitment **A, B.** qRT-PCR (A) and Western Blot (B) analyses of EMMPRIN expression in LLC cells used in this study. In both analyses, β-actin was used as an internal control. For qRT-PCR study, experiments were carried out in triplicate and the standard deviation is denoted using error bars. **C.** Growth curve of control LLC tumors (solid circle) and EMMPRIN^lo^-LLC tumors (open circle). Data is presented as the mean of tumor sizes and the standard error is denoted using error bars (*n* = 9). **D.** Average weights of control LLC tumors and EMMPRIN^lo^-LLC tumors at day 28 after inoculation. Representative images of a control LLC tumor (left) and an EMMPRIN^lo^-LLC tumor (right) are shown in the upper right corner. **E.** Average number of BMDC clusters per microscopic field in control LLC tumors and EMMPRIN^lo^-LLC tumors. Representative images of GFP-expressing BMDC cluster in control LLC tumors (left) and EMMPRIN^lo^-LLC tumors (right) are shown in the upper right corner. Cell nuclei were stained with DAPI. Standard deviation is denoted using error bars (*n* = 27). * represents *P* < 0.05. ** represents *P* < 0.001.

To enable spatial detection of BMDCs in mice, we generated C57BL/6 mice with GFP-labeled BMDCs through bone marrow transplantation. Four weeks after transplantation, mice were randomly grouped and received either inoculation of EMMPRIN^lo^-LLC cells or control-LLC cells, which were generated through transduction with an artificial lentivirus containing a well-characterized EMMPRIN target sequence or a scramble sequence, respectively. The down-regulation of EMMPRIN in EMMPRIN^lo^-LLC cells was confirmed using qRT-PCR (Figure 1A) and Western Blot (Figure 1B). After inoculation, the development of tumors was monitored. As expected, down-regulation of EMMPRIN significantly slowed tumor progression (Figure 1C). On day 28, mice were euthanized and the tumors were isolated. Western Blot analysis confirmed that the expression of EMMPRIN in EMMPRIN^lo^-LLC tumor tissues was lower than that in control LLC tumor tissues (Supplementary Figure S1). Results in Figure 1D show that the average weight of tumors in the experimental group was 41.7% less than that in the control group. Compared to control LLC tumors, tumors derived from EMMPRIN^lo^-LLC cells appeared to be significantly less vascularized. Next, tumors were sliced and subjected to microscopic examination for the presence of GFP^+^ BMDCs. We found that the average number of BMDCs per microscopic field in EMMPRIN^lo^-LLC tumors was 64% less than that in control LLC tumors (Figure 1E).

### Down-regulation of EMMPRIN reduces tumor metastasis, which is correlated with decreased BMDC clusters

**Figure 2:**
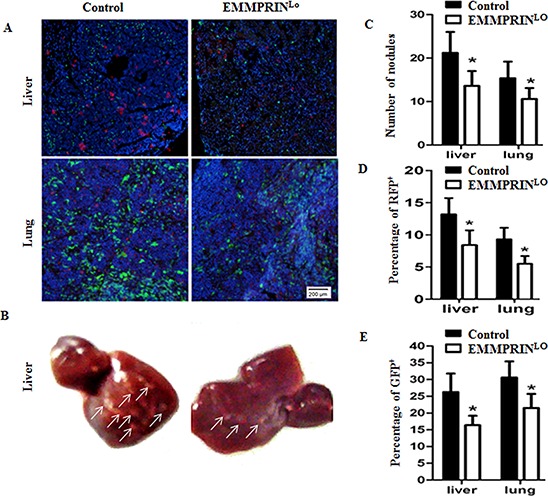
Down-regulation of EMMPRIN reduces tumor metastasis and BMDC recruitment **A.** Representative images of the livers (upper panel) and lungs (bottom panel) isolated from GFP^+^ BMDCs recipient mice with inoculation of red fluorescent protein (RFP)^+^ LLC cells or EMMPRIN^lo^-LLC. Blue signal represents DAPI nuclei staining, which identified all cells in the field. **B.** Representative images of the liver isolated from mice with inoculation of control LLC cells (left) and EMMPRIN^lo^-LLC cells (right). Tumor nodules are indicated by arrows. **C.** Average numbers of tumor nodules in the livers and lungs (*n* = 9). **D.** Average percentages of RFP^+^ LLC cells per microscopic field in the livers and lungs (*n* = 9). **E.** Average percentages of GFP^+^ BMDCs per microscopic field in the livers and lungs. The GFP or RFP positive area in a section was determined using ImageJ Software and shown as a percentage of the total section area. For each animal, the percentage of GFP or RFP positive area of three sections was counted and averaged. Data is presented as the mean and the standard error is denoted using error bars (*n* = 27). Filled bars and open bars represented mice with inoculation of control LLC cells and EMMPRIN^lo^-LLC cells, respectively. * represents *P* < 0.05.

We evaluated the role of EMMPRIN in tumor metastases. To allow detection of metastatic tumor cells in organs, we engineered LLC cells to express red fluorescence protein (RFP) through lentiviral transduction and implanted them into the left flanks of recipient mice with GFP^+^ BMDCs. Twenty-eight days later, mice were euthanized. The livers and lungs were harvested, sliced, and subjected to microscopic examination. Consistent with previous report [22], we detected both tumor cells, indicated by RFP signal, and BMDCs, indicated by GFP signal, in the livers and lungs of sacrified mice (Figure 2A).

We next investigated the impact of down-regulation of EMMPRIN on the formation of metastatic tumors and BMDC clusters. RFP-expressing EMMPRIN^lo^-LLC cells and control LLC cells were generated and implanted into the left flanks of recipient mice with GFP^+^ BMDCs. Twenty-eight days later, mice were euthanized and the livers and lungs were isolated. The presence of tumor cells and BMDCs was analyzed. We found that mice inoculated with EMMPRIN^lo^-LLC cells had significantly fewer tumor nodules in both the livers and lungs, which were 64.7% and 66.4% of those mice inoculated with control LLC cells, respectively (Figure 2C). Representative images of the liver are shown in Figure 2B. In accordance with this finding, microscopic examination of the liver and lung slices demonstrated that in comparison to mice inoculated with control cells, mice inoculated with EMMPRIN^lo^-LLC cells had significantly fewer of RFP^+^ LLCs (Figure 2D) and GFP^+^ BMDCs (Figure 2E).

### Down-regulation of EMMPRIN decreases tumor vascularization

**Figure 3:**
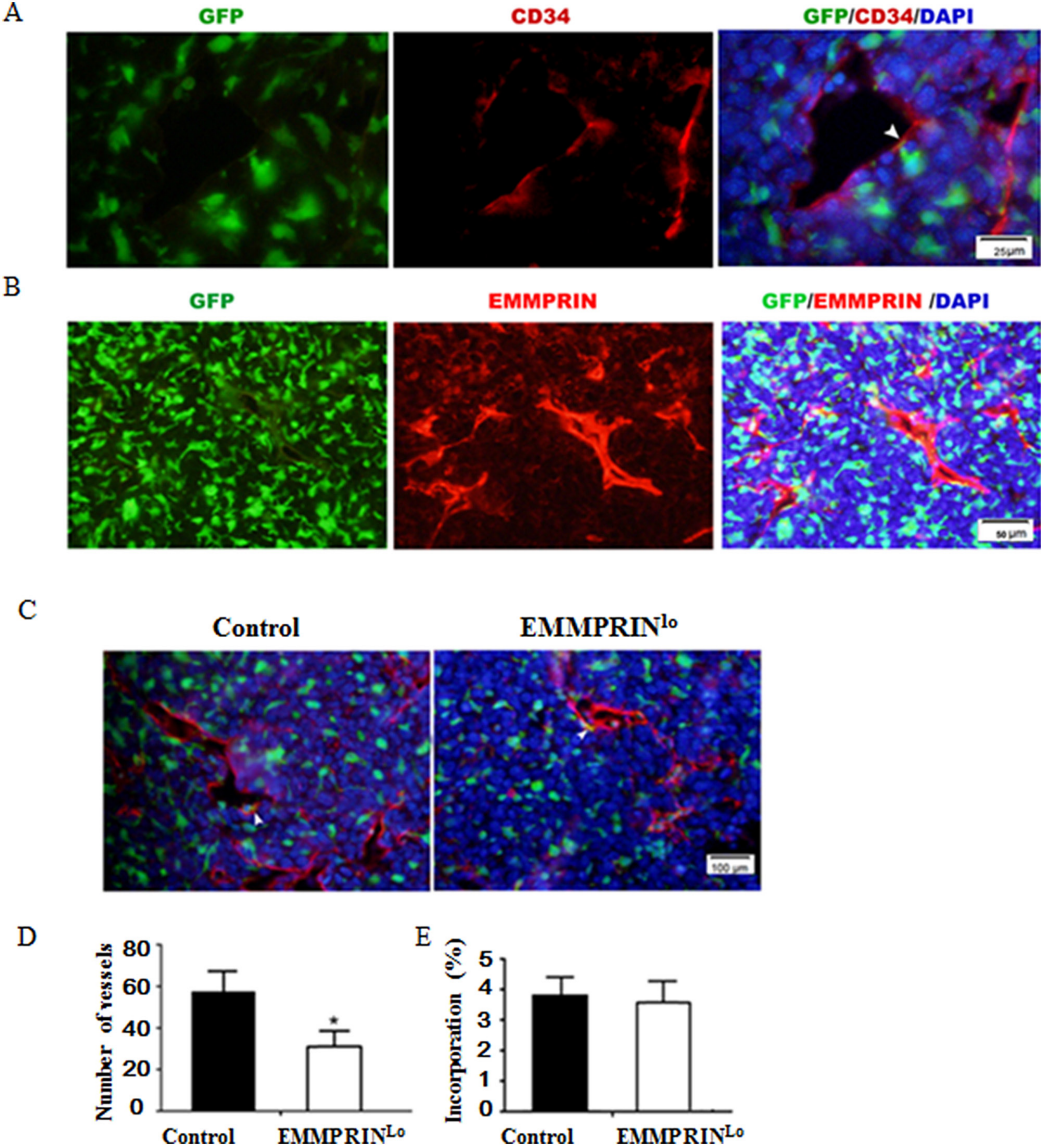
Down-regulation of EMMPRIN decreases tumor vascularization **A.** Representative immunofluorescence images of tumor sections stained with CD34 antibody. Green and red represent BMDCs and CD34^+^ vessel cells, respectively. **B.** Representative immunofluorescence images of tumor sections stained with EMMPRIN antibody. Red represented EMMPRIN^+^ cells. **C.** Representative images of a control LLC tumor (left) and EMMPRIN^lo^-LLC tumor (right). Green and red represent BMDCs and CD34^+^ vessel cells, respectively. EMMPRIN and CD34 are cell surface molecules. In A-C, EMMPRIN and CD34 stained endothelial cells which were well aligned with blood vessels. As such, both EMMPRIN and CD34 staining appeared to be linear and not clearly outline cells. **D.** Average numbers of vessels in tumors per microscopic field. **E.** Average percentages of BMDCs incorporated into neovessels per microscopic field. In the tumor slides with CD34 antibody staining, neovessel cells were stained red and BMDCs were labeled green with GFP. Only these green BMDCs cells co-localized with red vessel cells are counted as “incorporated BMDCs”. Data is presented as the mean and the standard deviation is denoted using error bars (*n* = 27). * represents *P* < 0.05.

To determine the role of BMDCs in tumor development, we stained the tumor slices with an antibody targeting CD34, a vascular endothelial precursor cell marker. Results in Figure 3A demonstrates that a small portion of GFP^+^ BMDCs aligned with the luminal side of vessels, suggesting that they were incorporated into tumor neovessels and contributed to tumor neovascularization. We further stained tumor slices with EMMPRIN antibody and found that EMMPRIN was also highly expressed on the luminal side of these blood vessels (Figure 3B) and colocalized with BMDCs (Figure 3B and Supplementary Figure S2). In particular, the density of blood vessels in EMMPRIN^lo^-LLC tumors was significantly lower than that in the control LLC tumors (Figure 3C). Quantitatively, EMMPRIN^lo^-LLC tumors had 34.4% fewer blood vessels than that in control LLC tumors (Figure 3D), although there was no significant difference in the percentage of BMDC incorporation between EMMPRIN^lo^-LLC tumors and control LLC tumors (Figure 3E). In our previous studies using human umbilical vein endothelial cells (HUVECs) as a model, we demonstrated that EMMPRIN regulates tumor vascularization [13, 23]. Together with these studies, the finding of a low density of blood vessels in EMMPRIN^lo^-LLC tumors suggests that the role of BMDCs in tumor vascularization may be regulated by EMMPRIN.

### EMMPRIN recruits BMDCs through paracrine signaling of SDF-1 and VEGF

**Figure 4:**
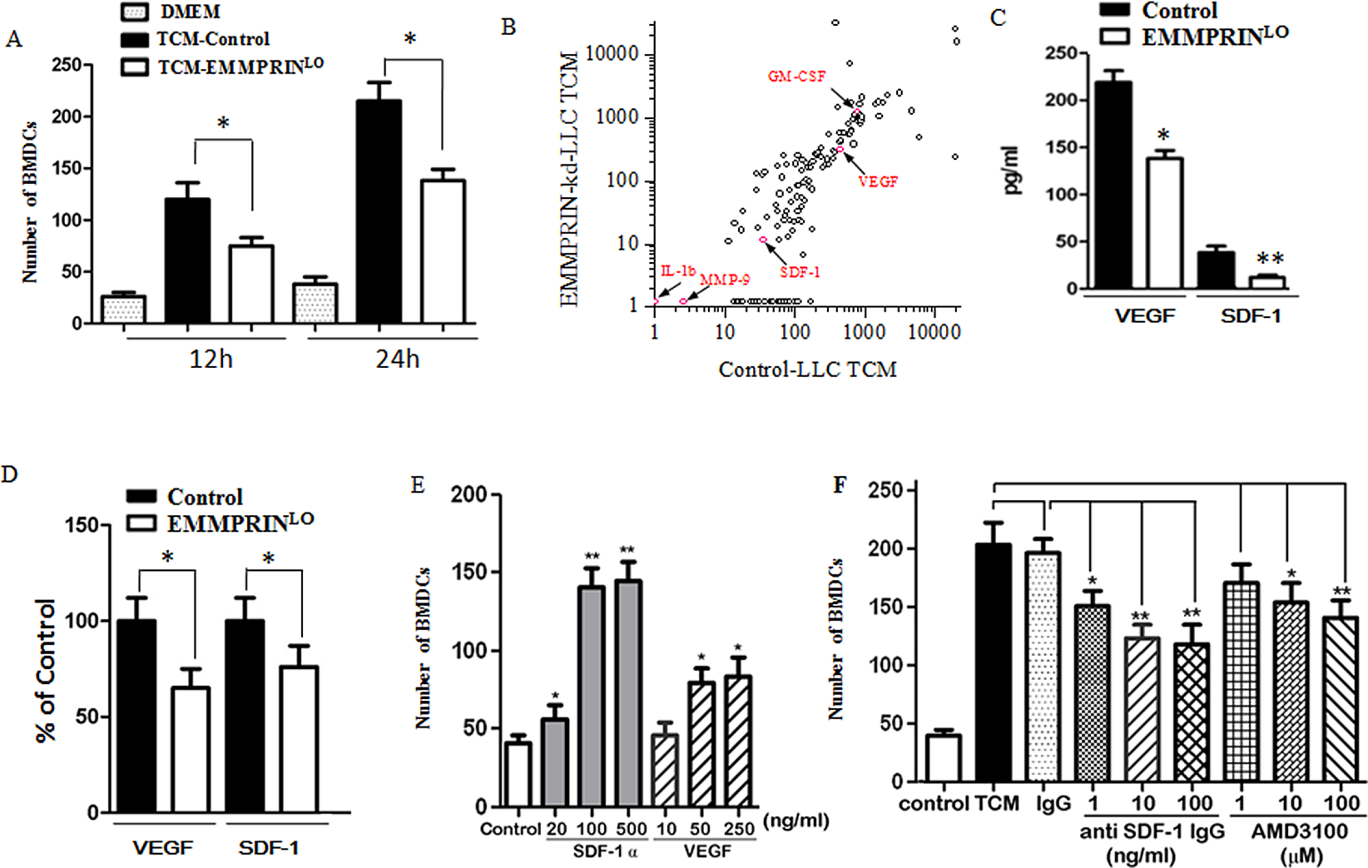
Chemotactic profile by antibody arrays **A.** Chemotactic effects of TCM on BMDCs. Down-regulation of EMMPRIN reduces the chemotactic effect of TCM. **B.** Profiles of soluble factors in control LLC TCM and EMMPRIN^lo^-LLC TCM. Red dots represent the proteins discussed in the manuscript. Other dots without labeling represent the rest of proteins determined by the antibody array. **C.** Quantification of VEGF and SDF-1 in control LLC TCM and EMMPRIN^lo^-LLC TCM by ELISA. **D.** qRT-PCR analysis of VEGF and SDF-1 expression in LLC and EMMPRIN^lo^-LLC. Sequences of primer were listed in Supplementary Table S3. **E.** Chemotactic effects of VEGF and SDF-1 on BMDCs. **F.** The chemotactic effect of SDF-1 is reduced by an SDF-1 antibody or AMD3100, an antagonist of the SDF-1 receptor, CXCR4. * represents *P* < 0.05. ** represents *P* < 0.001.

Our results suggest that EMMPRIN regulates tumor growth and metastasis through recruitment of BMDCs. To investigate whether the recruitment of BMDCs is regulated by EMMPRIN in a paracrine manner, we evaluated the migration of BMDCs using the standard transwell migration assay. Tumor conditioned medium (TCM) from EMMPRIN^lo^-LLC cells and control LLC cells were used as chemoattractants. Results in Figure 4A suggest that, compared to plain DMEM medium, both TCMs stimulated BMDC migration, but EMMPRIN^lo^-LLC TCM demonstrated significantly less migration-stimulating capacity than control LLC TCM. These findings suggest that EMMPRIN^lo^-LLC TCM contained less chemotatic factors than control LLC TCM.

To identify the chemotatic factors responsible for stimulating BMDC migration, we profiled the soluble proteins in TCMs using a RayBio^®^ antibody array, which allows semi-quantification of 308 proteins simultaneously. The signal for each protein was captured and the background signal was subtracted. The signal cutoff was manually set at 10. Among the 308 proteins profiled, we found that 137 had signals greater than 10 in control LLC TCM (Figure 4B). The proteins evaluated and their corresponding signals in TCMs are presented in Supplementary Table S1. In general, EMMPRIN^lo^-LLC TCM contained less soluble factors than control LLC TCM. Sixty-five proteins were found to have greater than 2 fold difference between EMMPRIN^lo^-LLC TCM versus control LLC TCM. Some of these proteins such as axl and neurturine, have well known biological functions including cell growth and survival. Other proteins included adhesion receptors, such as ICAM-5 and selectin, tissue-remodeling factors, such as DKK-1 and MMP-2, and metabolic enzymes, such as adiponectin and insulysin, suggesting that EMMPRIN is a multifunctional regulator of tumor development. Well-characterized EMMPRIN-regulated proteins, including MMP-2 and VEGF, were significantly lower in EMMPRIN^lo^-LLC TCM, and were found to be 30%, and 70% of that in the control LLC TCM, respectively.

It was previously reported that cancer cells recruit BMDCs through secretion of soluble factors, including SDF-1 [24–26], VEGF [22, 27], MMP-9 [26], placental growth factor (PIGF) [22, 28], IL-1 beta [29], and granulocyte macrophage colony stimulating factor (GM-CSF) [30]. Among these, SDF-1, VEGF, MMP-9, IL-1 beta and GM-CSF were included in the array used in this study and, thus, their contributions to BMDC recruitment were examined based on the array results. We found that the signals of MMP-9 and IL-1 beta were low in both the control LLC TCM and the EMMPRIN^lo^-LLC TCM, suggesting that neither of these proteins is a major regulator of BMDC recruitment. However, SDF-1, VEGF, and GM-CSF showed strong signals in both TCMs. In contrast to GM-CSF, which had a greater signal in EMMPRIN^lo^-LLC TCM (1241 vs. 789), SDF-1 and VEGF had lower signals in EMMPRIN^lo^-LLC TCM. Upon down-regulation of EMMPRIN, the signals of SDF-1 and VEGF decreased by 69% (from 36 to 11) and 32% (from 449 to 304), respectively. The decrease in SDF-1 and VEGF in EMMPRIN^lo^-LLC TCM was confirmed at the protein level by an ELISA assay (Figure 4C) and at the transcription level by qRT-PCR (Figure 4D).

EMMPRIN^lo^-LLC TCM, which contained less of SDF-1 and VEGF, had a lower ability than control LLC TCM to stimulate BMDC migration. It is likely SDF-1 and VEGF are major chemotatic factors responsible for BMDC recruitment. We measured the chemotactic effects of SDF-1 and VEGF on BMDCs using the standard transwell migration assay. At identical concentrations, SDF-1 had significantly greater chemotactic effect on BMDCs than VEGF (Figure 4E). The chemotactic effect of VEGF on BMDCs has previously been studied in the LLC tumor model [22], and thus was not included in the remainder of the study. We found that the chemotactic effect of control LLC TCM could be significantly reduced by blocking the SDF-1–CXCR4 axis using an anti-SDF-1 antibody or CXCR4 antagonist, AMD3100 (Figure 4F), suggesting that SDF-1 may be an important factor for BMDC recruitment. However, treatment with either anti-SDF-1 or AMD3100 reduced BMDC migration by no more than 50%, suggesting that SDF-1 is not the sole chemoattractant in LLC TCM. The chemotactic effect of LLC TCM is most likely due to an additive or synergistic effect of SDF-1 and VEGF together.

### EMMPRIN regulates the expression and secretion of SDF-1

**Figure 5:**
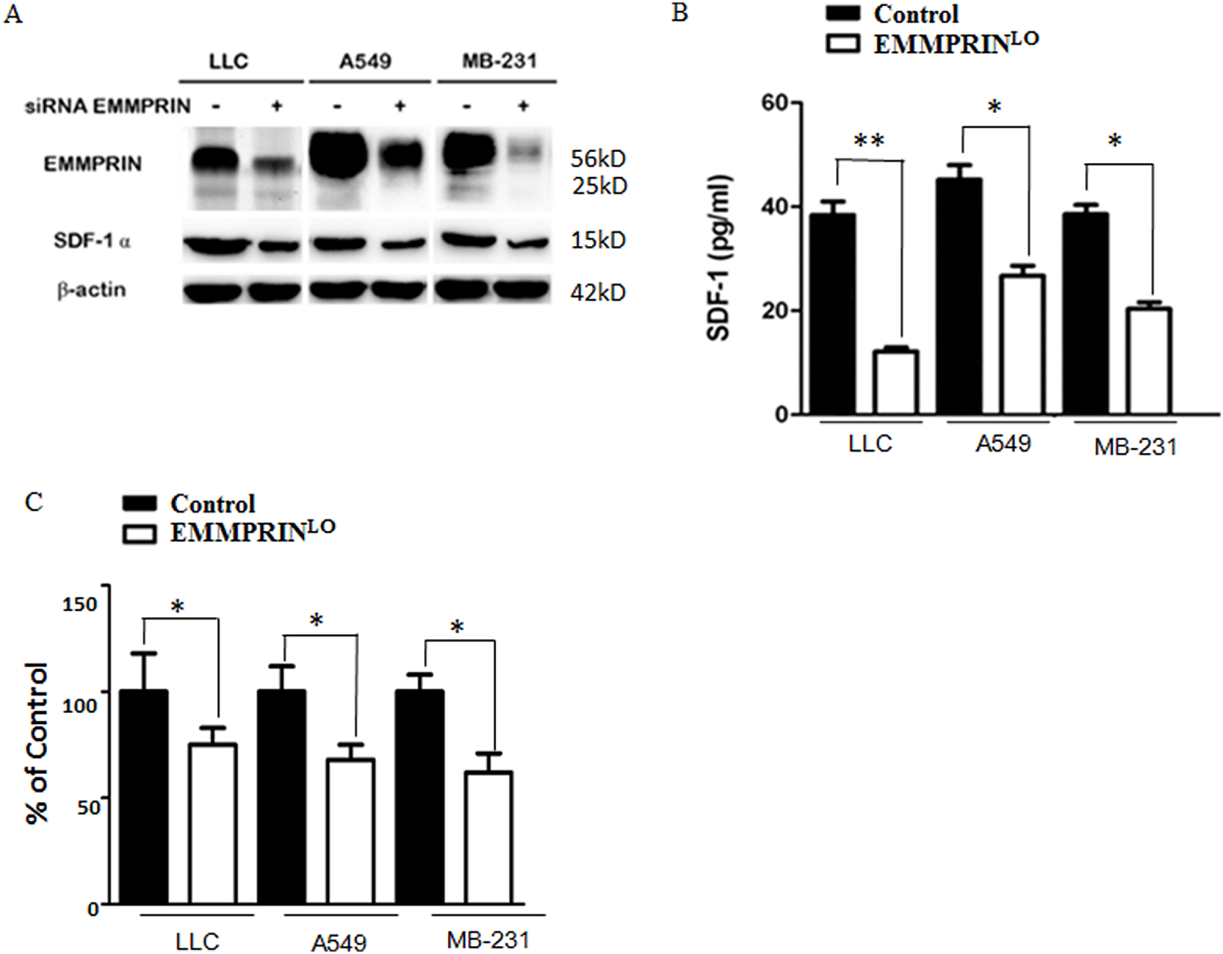
EMMPRIN controls the expression and secretion of SDF-1 **A.** Down-regulation of EMMPRIN decreases the expression of SDF-1 in LLC, A549 and MDA-MB-231 cells determined by Western Blot analysis. **B.** Down-regulation of EMMPRIN reduces the secretion of SDF-1 in LLC, A549 and MDA-MB-231 cells determined by ELISA analysis. **C.** Down-regulation of EMMPRIN reduces the expression of SDF-1 in LLC, A549 and MDA-MB-231 cells by qRT-PCR analysis. Sequences of primer were listed in Supplementary Table S3. Solid bars and open bars represent control cells and cells with down-regulation of EMMPRIN, respectively. * represents *P* < 0.05. ** represents *P* < 0.001.

The regulation of SDF-1 by EMMPRIN has not been previously reported. We explored whether the expression and secretion of SDF-1 are controlled by EMMPRIN. Using siRNAs, we knocked down the expression of EMMPRIN in LLC cells, A549 cells (human lung adenocarcinoma), and MDA-MB-231 cells (human breast carcinoma) and examined the expression of SDF-1 (Supplementary Table S2). We found that down-regulation of EMMPRIN significantly reduced the expression of EMMPRIN across all the three cell lines (Figure 5A). We also measured the level of SDF-1 protein in TCMs derived from these cell lines. In accordance with the decrease in SDF-1 protein expression, we found that down-regulation of EMMPRIN reduced the secretion of SDF-1 by 68.3, 40.9 and 47.2% in LLC cells, A549 cells, and MDA-MB-231 cells, respectively (Figure 5B). This trend was further confirmed at the transcription level by qRT-PCR (Figure 5C). Taken together, these results suggest that EMMPRIN controls the expression and secretion of SDF1.

## DISCUSSION

Previous studies have revealed that several signaling pathways involved in tumor growth and metastasis are regulated by EMMPRIN. These include pathways involved in the regulation of MMPs [8, 9], VEGF [10, 11], uPA system [14], and hyaluronan [15]. We also recently reported that EMMPRIN promotes tumor glycolysis through MCTs [12], and regulates tumor angiogenesis through IGF-I and VEGF [13]. However, the impact of EMMPRIN on BMDCs or on crosstalk between tumor cells and BMDCs has not been investigated. In the present study, by studying GFP-expressing BMDC donor cells in LLC tumor xenografts, we found that EMMPRIN regulates tumor growth and metastasis through recruitment of BMDCs, which contribute to tumor neovascularization. Further protein profiling studies revealed that EMMPRIN controlled BMDC recruitment by regulating the secretion of soluble factors, including VEGF and SDF-1.

The crosstalk between tumor cells and BMDCs in the LLC tumor model has been previously studied by Kaplan and colleagues [22]. They reported that VEGFR1^+^ BMDCs represent the major cellular population initiating the pre-metastatic niche, suggesting that the VEGF/VEGFR1 axis is the predominant signaling pathway between BMDCs and LLC cells in the pre-metastatic stage. Our study suggested that in addition to the VEGF/VEGFR1 axis, tumors and BMDCs also interact through the SDF-1/CXCR4 axis. The critical role of SDF-1 in BMDC recruitment has been previously demonstrated in a variety of tumor [24–26] and ischemia models [31–33]. Therefore, although it may not be critical for the formation of pre-metastatic niche, the SDF-1/CXCR4 axis is important for the recruitment of BMDCs to tumor microenvironment. Further studies that evaluate and compare the contribution of the VEGF/VEGFR1 axis and the SDF-1/CXCR4 axis in EMMPRIN-mediated BMDC recruitment are warranted.

The contribution of the SDF-1/CXCR4 axis to lung cancer development and metastasis identified in this study is consistent with previous reports by others, in which the SDF-1/CXCR4 axis was found to be associated with lung cancer metastasis [34–37] as well as patient survival [34, 36]. Due to its pivotal role, the SDF-1/CXCR4 axis represents a promising target for lung cancer treatment. Indeed, in preclinical studies, it was demonstrated that neutralization of SDF-1 by an anti-SDF-1 or anti-CXCR4 monoclonal antibody resulted in a significant decrease of lung cancer metastases [38, 39].

Taken together, our study reveals a novel mechanism by which EMMPRIN promotes tumor growth and metastasis through recruitment of BMDCs. Due to its pleiotropic effects, EMMPRIN represents a promising target for future antineoplastic therapy.

## MATERIALS AND METHODS

### Cell culture

LLC cells, A549 cells, MDA-MB-231 cells and HEK293 cells were obtained from American Type Culture Collection or Shanghai Institutes for Biological Sciences and were cultured in DMEM medium supplemented with 10% fetal bovine serum, 100 units/mL penicillin, and 100 μg/mL streptomycin (Invitrogen) at 37°C.

LLC cells with down-regulation of EMMPRIN, EMMPRIN^lo^-LLC cells, and control LLC cells, were generated through lentiviral transduction. EMMPRIN target sequence (5′-GAGGCAAUCACCAAUAGCATT-3′, 5′-UGCUAUUGGUGAUUGCCUCTT-3′) and control scramble sequence (5′-GAAGCAGCACGACUUCUUCT T-3′/5′-GAAGAAGUCGUGCUGCUUCTT-3′) were synthesized and cloned into the Age I and EcoR I restriction endonuclease sites of the lentiviral vector pMAGic 4.1. The target sequences have been characterized in a previous study. [40] Artificial lentiviruses were generated in HEK293 cells through transfection with a selected pMAGic construct together with pMD2.G (Addgene #12259) and psPAX2 (Addgene #12260) according to the standard protocol. Stable cell lines were selected and maintained in medium with 1 mg/ml neomycin (Sigma-Aldrich). Gene silencing was confirmed by quantitative RT-PCR (qRT-PCR) and Western Blot as described previously [13].

For experiments described in Figure 5, down-regulation of EMMPRIN was achieved through transfection with siRNAs (Ambion, Austin, TX) using lipofectamine RNAiMAX (Invitrogen) and confirmed by Western Blot. siRNA duplexes were purchased from Ambion Inc. (Austin, TX). EMMPRIN target sequences are described in Supplementary Table S2.

### BMDC isolation and transplantation

All procedures were performed under the guidelines of the Institutional Animal Care and Use Committee. Wild type and green fluorescent protein (GFP) transgenic C57BL/6 mice were obtained from the Animal Center of Fourth Military Medical University and were maintained in a germ-free environment with food and water access. BMDCs were harvested from 6 to 12-week-old GFP mice by flushing the femurs and tibias with 2% FBS in Hank's balanced salt solution (Invitrogen). For BMDC transplantation, recipient C57BL/6 mice were lethally irradiated (950 rads) and received intravenous administration of GFP^+^ BMDCs via the lateral tail vein.

### Tumor implantation and monitoring

Four weeks after BMDC transplantation, mice were randomly grouped into two groups, receiving either subcutaneous inoculation of 2 × 10^6^ WT-LLCs or EMMPRIN^lo^-LLCs, respectively. Mice were closely monitored and the tumor sizes were measured using a vernier caliper every four days. The tumor volume was calculated using the following formula: *V* = *lw*^2^/2. The growth curves were plotted using the mean of the tumor volumes for each treatment group at a given time point. Four weeks after tumor inoculation, mice were euthanized. The tumors were removed and subjected to analysis.

### Immunohistochemistry

In the study evaluating the contribution of BMDCs and EMMPRIN to tumor vascularization, mice were euthanized 4 weeks after tumor implantation. The livers and lungs were collected and fixed in 4% paraformaldehyde. After soaking in 10% sucrose for 1 h and 30% sucrose at 4°C overnight, the livers and lungs were embedded in optimum cutting temperature (OCT) embedding compound (McCormick, USA) and sliced with a freezing microtome (Leica, Germany). Sections 10 μm in thickness were obtained and stained using rabbit anti-mouse CD34 (ABCAM) and rabbit anti-mouse EMMPRIN antibody (ABCAM), followed by Alexa Fluor 594-labeled goat anti-rabbit IgG secondary antibody (Invitrogen). Slices were mounted with Vectashield medium with DAPI (Vector Laboratories) and subjected to microscopic examination using a laser scanning confocal microscope (Olympus, Japan).

### Transwell migration assay

Transwell migration assay was performed using 24-well migration Boyden chambers with polycarbonate filters (pore size, 8 μm, Millipore). Experiments were carried out according to the standard procedures. Briefly, TCM was added to the lower chamber prior to the addition of insert as chemoattractant. Next, an insert was placed into the chamber and BMDCs (3 × 10^5^ cells/well) in DMEM medium without supplements were added to the upper compartment of the insert. Twelve or 24 hours after incubation, non-migrated cells in the upper chamber were removed using a cotton swab. Migrated cells on the bottom of membrane were fixed in 100% methanol and stained with 0.5% crystal violet in 2% ethanol. Photographs were taken randomly using at least five fields for each membrane using a microscope. The number of migrated cells was expressed as the average number of cells per microscopic field over five different fields. All assays were performed in triplicate.

For the study evaluating the role of SDF-1 in BMDC migration, SDF-1 in TCM was eliminated through immunoprecipitation. Briefly, TCM was incubated with goat anti-SDF-I neutralizing antibody (R&D System) at 1, 10, or 100 ng/ml at 4°C for 12 h, followed by incubation with rabbit anti-goat IgG at 1 ug/ml (Thermo Scientific) and Protein A Sepharose beads (Sigma) at 4°C overnight. Beads were removed by centrifugation and the supernatant was collected. The reduction of SDF-1 in TCM was confirmed using an ELISA assay kit (ABCAM) according to the manufacturer's instructions. The effect of blocking the SDF-1/CXCR4 axis was also studied by treating BMDCs with CXCR4 inhibitor, AMD3100, at 1, 10, and 100 μM (Sigma).

### Chemotactic profile by antibody array

The secretion of soluble factors by LLC cells was evaluated in duplicate using a protein array method (RayBio^®^Biotin label-based Mouse Antibody Array#AAM-BLG2000, RayBiotech). This assay is capable of simultaneously detecting 308 different proteins with high specificity. Briefly, TCM media was obtained after the incubation of 2 × 10^5^ cells in 1.5ml serum-free medium for 20 h at 37°C and 5% CO_2_. To determine the relative concentrations of soluble factor in media, the density of each individual spot was measured using ImageJ software (National Institutes of Health).

### ELISA assay

The levels of SDF-1 and VEGF in TCM were determined by using ELISA assay kits from ABCAM. Experiments were carried out according to the manufacturer's instructions. Absorbance was acquired using a VersaMax Tunable MicroPlate Reader (Molecular Devices, Sunnyvale, CA) at 490 nm.

### Statistical analysis

Differences in different groups were compared using the unpaired Student's *t*-test using SigmaPlot 12.0.

## SUPPLEMENTARY FIGURES AND TABLES


