# Supplementary material for: EMMPRIN regulates tumor growth and metastasis by recruiting bone marrow-derived cells through paracrine signaling of SDF-1 and VEGF
Source: Oncotarget. 2015 Sep 22;6(32):32575–85. doi: 10.18632/oncotarget.5331 (PMC4741713; doi:10.18632/oncotarget.5331)
Supplement: Supplementary file 1 [file oncotarget-06-32575-s001.pdf]

SUPPLEMENTARY FIGURES AND TABLES

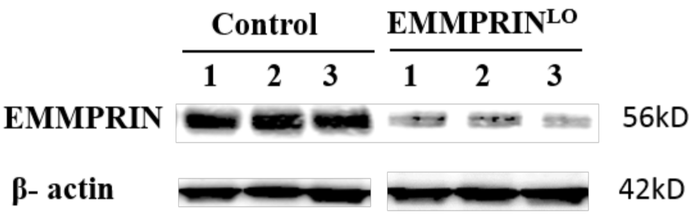

Supplementary Figure S1: Western Blot analysis of EMMPRIN expression in tumor tissues. Three reprehensive tumors from each group were isolated from mice and subjected to Western Blot as described in Materials and Methods.

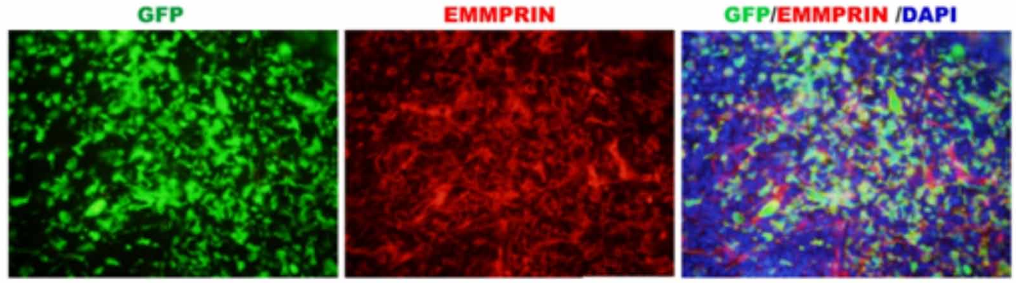

Supplementary Figure S2: Immunofluorescence images of tumor sections stained with anti-EMMPRIN antibody. Green and red represent BMDCs and EMMPRIN^{+} cells, respectively. As shown in these images, EMMPRIN expression (red) is colocalized with BMDCs (green), resulting in yellow signals.

Supplementary Table S1: Proteins and their signals detected by the RayBio® arrays. Only those proteins with over 2 fold change, either greater or smaller, are listed.

Protein	LLC-Control	LLC-EMMPRIN^{Lo}	LLC-EMMPRIN^{Lo}/ LLC-Control
Adiponectin / Acrp30	69.77	246.12	3.53
Artemin	35.01	123.31	3.52
Axl	1	119.17	119.17
Chordin-Like 2	37.65	133.68	3.55
Coagulation Factor III	28.41	128.5	4.52
CTACK	160.85	79.28	0.49
CTLA-4 /CD152	112.01	253.89	2.27
CXCL16	649.69	1737.84	2.67
CXCR2 / IL-8 RB	60.53	11.4	0.19
DAN	36.77	1.18	0.03
Dkk-1	4709.13	1261.67	0.27
Dkk-4	1	14.51	14.51
EGF R	100.57	21.76	0.22
Eotaxin / CCL11	1	129.54	129.54

(Continued)

Protein	LLC-Control	LLC-EMMPRIN^{Lo}	LLC-EMMPRIN^{Lo}/ LLC-Control
FGF-21	1	54.4	54.4
Flt-3 Ligand	8.17	1.18	0.14
Frizzled-1	14.77	1.18	0.08
Frizzled-7	56.57	166.84	2.95
GDF1	15.21	1.18	0.08
GITR / TNFRF18	133.13	6.73	0.05
ICAM-2	26.65	1.18	0.04
ICAM-5	108.05	1.18	0.01
IGFBP-1	410.77	1470.48	3.58
IGFBP-rp1 / IGFBP-7	6091.17	486.53	0.08
IL-1 RI	179.77	16.58	0.09
IL-1 RII	71.09	1.18	0.02
IL-2 R alpha	75.93	1.18	0.02
IL-2 R beta	15.88	1.18	0.07
IL-6 R	178.45	72.54	0.41
IL-9	95.29	15.54	0.16
IL-10 R alpha	100.57	1.18	0.01
IL-10 R beta	15.21	1.18	0.08
IL-12 p40	112.45	1.18	0.01
IL-12 p70	20164	235.75	0.01
IL-17	61.41	1.18	0.02
IL-17D	170.97	1.18	0.01
IL-18 R alpha /IL-1 R5	17.41	1.18	0.07
IL-20	112.01	32.64	0.29
IL-20 R alpha	138.85	47.67	0.34
IL-22 BP	63.17	1.18	0.02
IL-28	27.97	72.02	2.58
I-TAC / CXCL11	117.73	23.83	0.2
Kremen-1	77.25	33.16	0.43
LRP-6	86.05	23.83	0.28
L-Selectin (CD62L)	73.29	27.46	0.37
MFG-E8	44.69	1.18	0.03
MIP 2	625.93	7043.59	11.25
MMP-2	71.6	23.92	0.33
MMP-3	50.41	1.18	0.02

(*Continued*)

Protein	LLC-Control	LLC-EMMPRIN^{Lo}	LLC-EMMPRIN^{Lo}/ LLC-Control
MMP-9	2.57	1.18	0.46
Neurturin	50.41	1.18	0.02
NOV / CCN3	31.49	1.18	0.04
Osteoactivin / GPNMB	64.05	1.18	0.02
Osteocrin	28.41	1.18	0.04
Prolactin	78.13	178.75	2.29
SCF	126.09	22.28	0.18
SDF-1 / CXCL12	35.8	11.29	0.32
SIGIRR	57.89	1.18	0.02
SLPI	935.25	2100.02	2.25
Spinesin	18.29	1.18	0.06
TCCR / WSX-1	7.73	1.18	0.15
TECK / CCL25	5.97	1.18	0.2
TGF-beta RI / ALK-5	386.57	31164	80.62
TIMP-1	56.13	19.18	0.34
TMEFF1 / Tomoregulin-1	47.77	1.18	0.02
TNF RI / TNFRSF1A	24.01	1.18	0.05
TREM-1	84.29	1.18	0.01
Ubiquitin	126.97	53.37	0.42
uPAR	64.49	1.18	0.02
VE-Cadherin	13.45	1.18	0.09
VEGF-D	37.65	1.18	0.03
WISP-1 / CCN4	5.97	1.18	0.2

Supplementary Table S2: Sequences of siRNA used in the study

Target gene	Sequences
Mouse EMMPRIN-1	5′-CGUAGAUUCCCAUCAUACAdTdT-3′;<br>5′-UGUAUGAUGGGAAUCUACGdGdG-3′
Mouse EMMPRIN-2	5′GGAUACAGGUCGGAAAGAAdTdT-3′<br>5′UUCUUUCCGACCUUGAUCCdTdG-3′
Mouse EMMPRIN-3	5′-GAGGCAAUCACCAAUAGCAdTdT-3′<br>5′-UGCUAUUGGUGAUUGCCUCdTdT-3′
Human EMMPRIN	5′-GUUCUUCGUGAGUUCCUCdTdT-3′<br>5′GAGGAACUCACGAAGAACdTdT-3′
Scrambled Control	5′-GAAGCAGCACGACUUCUUCdTdT-3′<br>5′-GAAGAAGUCGUGCUGCUUCdTdT-3′

Supplementary Table S3: Sequences of primer used in the study

Target gene	Sequences
Mouse EMMPRIN	5′-CCGGCCAGAAAACGGACTT-3′;<br>5′-AGGAAGACGCAGGAGTACTCT-3′
Mouse SDF-1	5′-GAAGTGGAGCCATAGTAATGCC-3′<br>5′-TCCAAGTGGAAAAATACACCG-3′
Mouse VEGF	5′-TGGATGTCTACCAGCGAAGC-3′<br>5′-ACAAGGCTCACAGTGATTTT-3′
Mouse GAPDH	5′-GCCAAGGTCATCCATGACAACTTTGG-3′<br>5′GCCTGCTTCACCACCTTCTTGATGTC-3′
Human EMMPRIN	5′-TCGCGCTGCTGGGCACC-3′<br>5′-TGGCGCTGTCATTCAAGGA-3′
Human SDF-1	5′-AGTCAGGTGGTGGCTTAACAG-3′<br>5′-AGAGGAGGTGAAGGCAGTGG-3′
Human VEGF	5′-CTCACCGCCTCGGCTTGTCACA-3′<br>5′-CCTGGTGGACATCTTCCAGGAGTA-3′
Human GAPDH	5′-GGTGGTCTCCTCTGACTTCAACA-3′<br>5′-GTTGCTGTAGCCAAATTCGTTGT-3′
